# APOBEC Alteration Contributes to Tumor Growth and Immune Escape in Pan-Cancer

**DOI:** 10.3390/cancers14122827

**Published:** 2022-06-08

**Authors:** Honghong Guo, Ling Zhu, Lu Huang, Zhen Sun, Hui Zhang, Baoting Nong, Yuanyan Xiong

**Affiliations:** Key Laboratory of Gene Engineering, Ministry of Education, Institute of Healthy Aging Research, School of Life Sciences, Sun Yat-Sen University, Guangzhou 510006, China; guohh7@mail2.sysu.edu.cn (H.G.); zhuling8@ivf-cq.com.cn (L.Z.); huanglu705122613@foxmail.com (L.H.); sunzh39@mail2.sysu.edu.cn (Z.S.); zhangh357@mail2.sysu.edu.cn (H.Z.); nongbt@mail2.sysu.edu.cn (B.N.)

**Keywords:** APOBEC, pan-cancer, genetic alterations, tumor microenvironment, survival, immunotherapy, clinical relevance

## Abstract

**Simple Summary:**

The APOBEC3 family (apolipoprotein B mRNA editing enzyme catalytic polypeptide-like) was shown to induce tumor mutations through an aberrant DNA editing mechanism. In this study, we found that APOBEC genes were widely and significantly differentially expressed between normal and cancer samples in 16 cancer types, and their expression levels were significantly correlated with the prognostic value in 17 cancer types. Further analysis of the APOBEC family revealed extensive regulatory mechanisms by which they affect the tumor microenvironment, the process of tumor oncogenesis and development, and their association with patient prognosis in pan-cancer.

**Abstract:**

The accumulating evidence demonstrates that the apolipoprotein B mRNA editing enzyme catalytic polypeptide-like (APOBEC), DNA-editing protein plays an important role in the molecular pathogenesis of cancer. In particular, the APOBEC3 family was shown to induce tumor mutations by an aberrant DNA editing mechanism. However, knowledge regarding the reconstitution of the APOBEC family genes across cancer types is still lacking. Here, we systematically analyzed the molecular alterations, immuno-oncological features, and clinical relevance of the APOBEC family in pan-cancer. We found that APOBEC genes were widely and significantly differentially expressed between normal and cancer samples in 16 cancer types, and that their expression levels are significantly correlated with the prognostic value in 17 cancer types. Moreover, two patterns of APOBEC-mediated stratification with distinct immune characteristics were identified in different cancer types, respectively. In ACC, for example, the first pattern of APOBEC-mediated stratification was closely correlated with the phenotype of immune activation, which was characterized by a high immune score, increased infiltration of CD8 T cells, and higher survival. The other pattern of APOBEC-mediated stratification was closely correlated with the low-infiltration immune phenotype, which was characterized by a low immune score, lack of effective immune infiltration, and poorer survival. Further, we found the APOBEC-mediated pattern with low-infiltration immune was also highly associated with the advanced tumor subtype and the CIMP-high tumor subtype (CpG island hypermethylation). Patients with the APOBEC-mediated pattern with immune activation were more likely to have therapeutic advantages in ICB (immunological checkpoint blockade) treatment. Overall, our results provide a valuable resource that will be useful in guiding oncologic and therapeutic analyses of the role of APOBEC family in cancer.

## 1. Introduction

APOBEC (apolipoprotein B mRNA editing enzyme, catalytic polypeptide-like) genes are cytidine deaminases that deaminate cytidine to uridine in DNA and RNA [1,2]. In the normal repair process of APOBEC deamination, cytosine residue in the DNA sequences is restored again by the removal of uracil by DNA glycosylase and by replacing the cytosine in the abasic site, however, the abasic site is occasionally replaced with thymine or guanine. This single base substitution will result in a mutation in the genome [3,4,5,6]. It was reported that the intrinsic DNA editing capability of APOBEC enzymes is correlated with diverse vital biological processes, including immunity, antigen presentation, metabolism, regulation of gene expression, and maturation of host immune receptors [7,8]. Since the specific recognition of cytosine on single-stranded DNA depends on its adjacent base sequence during the process of APOBEC deamination, APOBEC-mediated DNA mutation has to leave a unique APOBEC mutation signature, and each member of the APOBEC family has its own specific mutation signature [9,10,11,12]. Moreover, the APOBEC mutation signature is the most universal pathological mutagenesis mechanism, and the APOBEC mutation signature was detected in at least 22 different tumor types and is enriched in bladder, head and neck, cervical, and breast cancers [13,14]. In particular, more than half (16/30) of cancer types have *APOBEC3B*-mediated mutation signatures [1]. In addition, increasing evidence demonstrated that APOBEC genes are often deregulated in cancers, and the DNA damage caused by the aberrant activity of the APOBEC enzyme was the major driving force behind iterative somatic mutation across many cancer types [3,15]. Therefore, the APOBEC family plays a crucial role in driving cancer genome instability [8], promoting intratumor heterogeneity and promoting the divergence in the genome, which often results in many subclones evolving with drug resistance and immune-escape capacity [16].

A total of 11 APOBEC genes are present in the human genome. *AICDA* and *APOBEC1* are on chromosome 12, *APOBEC4* is on chromosome 1, and *APOBEC2* is on chromosome 6. *APOBEC3* gene cluster, comprising of seven members (*APOBEC3A*, *APOBEC3B*, *APOBEC3C*, *APOBEC3D*, *APOBEC3F*, *APOBEC3G*, and *APOBEC3H*), reside at chromosome 22. Among them, four APOBEC genes were considered to participate in biological functions for cancer, such as in the Cosmic database [17], the CancerMine database [18], and the TSGene database [19]; *APOBEC3A* was annotated as oncogenes, *APOBEC3B* was annotated as oncogenes and TSG (tumor suppressor gene), *APOBEC3G* was annotated as driver genes, and *AICDA* was annotated as oncogenes and driver genes. However, the relevant functional information of the other genes is lacking.

Immunotherapy was applied to cancer treatment and achieved better clinical outcomes, such as the immunological checkpoint blockade (ICB), however, it generally shows a low response rate. Numerous studies have shown that the tumor microenvironment (TME), on which tumor cells depend for growth and survival, plays a crucial role in the tumor progression immune escape, and its effect on response to immunotherapy. It is known that neoantigens (or neoepitopes) arise from missense somatic mutations in cancer cells [20]. Neoantigens that presented on the cell surface in the context of a major histocompatibility complex (MHC) of tumor tissues could be recognized by T cells as foreign antigens [21]. In a tumor microenvironment, a significant proportion of tumor-infiltrating lymphocytes that are comprised of immune cells, primarily from CD8^+^ cytotoxic T cells, was observed in many cancer types [22]. Thus, the APOBEC family may affect cancer immunogenicity by arousing neoantigens, and eventually affecting the therapeutic effect of ICB treatment. Recently, several studies revealed that the regulation of *APOBEC3B* in cancer can induce T cell responses by affecting neoepitopes [23,24,25], and is associated with a greater likelihood of response to immunotherapy response in non-small cell lung cancer [26], head and neck cancer, bladder cancer [27], breast cancer [25,28], and melanomas [23]. Nevertheless, the related research on other family members is relatively lacking. In addition, the anti-tumor effect is characterized by numerous tumor suppressor factors that interact in a highly coordinated manner, and the alteration of only one or two genes could not fully explain the complexity of the process. Thus, a comprehensive understanding of the genetic alterations and the perturbations in expression underlying cancer cell heterogeneity is necessary to elucidate the APOBEC regulation-based therapeutic targets.

In this study, we aimed to systematically characterize the molecular alterations, immuno-oncology features, and clinical relevance of the APOBEC family at the pan-cancer level. We found that the APOBEC family showed a relatively higher mutation rate in UCEC (uterine corpus endometrial carcinoma), and a relatively higher copy number variation of deletion frequency in MESO (mesothelioma) and OV (ovarian serous cystadenocarcinoma). Interestingly, there are extensively significant differences in the expression of APOBEC family between normal and cancer samples across cancer types (|log2FC| > 1, *p* < 0.05), and their expression levels are significantly correlated with the prognostic value (*p* < 0.05). Furthermore, we revealed two patterns of APOBEC-mediated stratification (AMS) with distinct TME infiltration characteristics, based on the 11 APOBEC gene expression profiles across cancer types, respectively. In ACC, for example, the first AMS pattern (namely, Cluster-A) was closely correlated with the phenotype of immune activation, which was characterized by a high immune score, increased infiltration of CD8 T cell, and better survival. The other AMS pattern (namely, Cluster-B) was closely correlated with the low-infiltration immune phenotype, which was characterized by a low immune score, lack of effective immune infiltration, and poorer survival. Further, we found that the pattern of AMS, with a low-infiltration immune phenotype, was also highly associated with the advanced tumor subtype and the CIMP-high tumor subtype (CpG island hypermethylation). Meanwhile, patients with the AMS pattern tumors with immune activation were more likely to have therapeutic advantages in ICB (immunological checkpoint blockade) treatment. Our analysis highlights the importance of the APOBEC family in cancer development and TME infiltration, and lays a foundation for the development of therapeutic strategies based on APOBEC regulation.

## 2. Materials and Methods

### 2.1. Genome-Wide Omics Data and Clinical Data across 33 Cancer Types

The somatic mutations (MAF file), copy number variation, RNA-seq data (counts and fragments per kilobase per million (FPKM) value), and clinical information for patients of 33 cancer types were downloaded from the TCGA project (http://cancergenome.nih.gov/ (accessed on 5 May 2021)) via the R package, TCGAbiolinks (version = 2.22.4) [29]. GISTIC2.0 [30] was used to identify the genomic regions that are significantly gained or lost. The FPKM values were transformed into transcripts per kilobase million (TPM) values. Clinical data downloaded by the R package TCGAbiolinks include the survival status, survival time, stages, histology subtype, gender, and race. Other clinical data, including tumor purity, TMB, and subtype information defined by previous studies, were collected from Bagaev et al. [31].

We analyzed 33 different TCGA cancer types in total, including ACC, adrenocortical carcinoma; BRCA, breast cancer; SKCM, skin cutaneous melanoma; KIRC, kidney renal clear cell carcinoma; LUSC, lung squamous cell carcinoma; KIRP, kidney renal papillary cell carcinoma; UCEC, uterine corpus endometrial carcinoma; KICH, kidney chromophobe; PRAD, prostate adenocarcinoma; BLCA, bladder urothelial carcinoma; STAD, stomach adenocarcinoma; COAD, colon adenocarcinoma; LUAD, lung adenocarcinoma; UVM, uveal melanoma; HNSC, head and neck squamous carcinoma; THCA, thyroid carcinoma; CESC, cervical squamous cell carcinoma and endocervical adenocarcinoma; LIHC, liver hepatocellular carcinoma; LGG, brain lower grade glioma; TGCT, testicular germ cell tumors; THYM, thymoma; GBM, glioblastoma multiforme; SARC, sarcoma; READ, rectum adenocarcinoma; UCS, uterine carcinosarcoma; PCPG, pheochromocytoma and paraganglioma; OV, ovarian serous cystadenocarcinoma; PAAD, pancreatic adenocarcinoma; ESCA, esophageal carcinoma; MESO, mesothelioma; DLBC, lymphoid neoplasm diffuse large b-cell lymphoma; LAML, acute myeloid leukemia; CHOL, cholangiocarcinoma.

Data were analyzed with the R (version 4.1) and R Bioconductor packages.

### 2.2. Identification of Differentially Expressed Genes (DEGs)

To explore the expression perturbations of the APOBEC family across cancer types, respectively, we performed differential expression analysis between TCGA normal and TCGA cancer samples across the 16 cancer types with at least 10 normal controls. The R package DESeq2 (version = 1.34.0) [32] was applied to determine differentially expressed genes (DEGs). The significance criteria for determining DEGs were set as |log2FC| > 1 and *p* < 0.05.

### 2.3. Unsupervised Clustering for 11 APOBEC Genes

The unsupervised clustering algorithm was applied to identify patients with qualitatively different patterns of APOBEC-mediated stratification based on the global expression pattern of 11 APOBEC genes across cancer types for further analysis. Each cancer type from TCGA was analyzed individually. The number of clusters and their stability were determined by the consensus clustering algorithm. We used the R package ConsensusClusterPlus (version = 1.58.0) to perform the above steps, with 1000 times repetitions to guarantee the stability of classification [33].

### 2.4. Estimation of TME Cell Infiltration

The ESTIMATE algorithm [34] was applied to the normalized expression matrix for estimating the immune scores for each cancer sample from TCGA by using R package estimate (version = 1.0.13). The abundance of 22 TME infiltrating immune cells was quantified in TCGA 33 cancer types, respectively, by using the CIBERSORT algorithm (https://cibersort.stanford.edu/ (accessed on 19 June 2021), a deconvolution algorithm that uses support vector regression to determine the type of immune cell type in tumors [35]. The parameters were as follows: the input mixture matrix was our normalized gene expression matrix, the input of gene signature reference for 22 immune cell types from Newman et al. [35], 100 times for the permutation test, and non-quantile normalization.

### 2.5. DEGs between Distinct Clusters and Functional Enrichment Analysis

To identify the two AMS patterns-related genes, we classified patients into two AMS patterns, based on the global expression of 11 APOBEC genes. R package DESeq2 (version = 1.34.0) [32] was applied, to determine the differentially expressed genes (DEGs) between different AMS patterns; the significance criteria for determining DEGs were set as |log2FC| > 1 and the FDR (discovery rate) < 0.05.

To further understand the pathways of different AMS patterns, gene ontology (GO) analysis of DEGs were performed by the R package clusterProfiler [36] (version = 4.2.2). Gene set enrichment analysis (GSEA) was utilized to analyze the functions related to AMS patterns, according to the comprehensive gene expression profiles [37]. Gene sets with *p* < 0.05 and FDR < 0.25 were considered significantly enriched.

### 2.6. Statistical Analysis

Correlation coefficients were computed by Pearson correlation analysis. One-way ANOVA tests were used to conduct difference comparisons of two groups [38]. The univariate and multivariate Cox analyses were performed to determine the independent risk characteristics. Hazard ratios (HRs) and 95% confidence intervals of these variables were estimated to quantify the strength of these associations. R package forestplot (version = 2.0.1, https://github.com/gforge/forestplot (accessed on 4 February 2022)) was employed to visualize the results of the multivariate prognostic analysis. The survival curves were generated via the Kaplan–Meier method and log-rank tests were utilized to identify the significance of differences by using the R package survival (version = 3.3.1). *p* < 0.05 were considered as significant.

## 3. Results and Discussion

### 3.1. The Landscape of Genetic Alteration of APOBEC Family across Cancer Types

**Figure 1:**
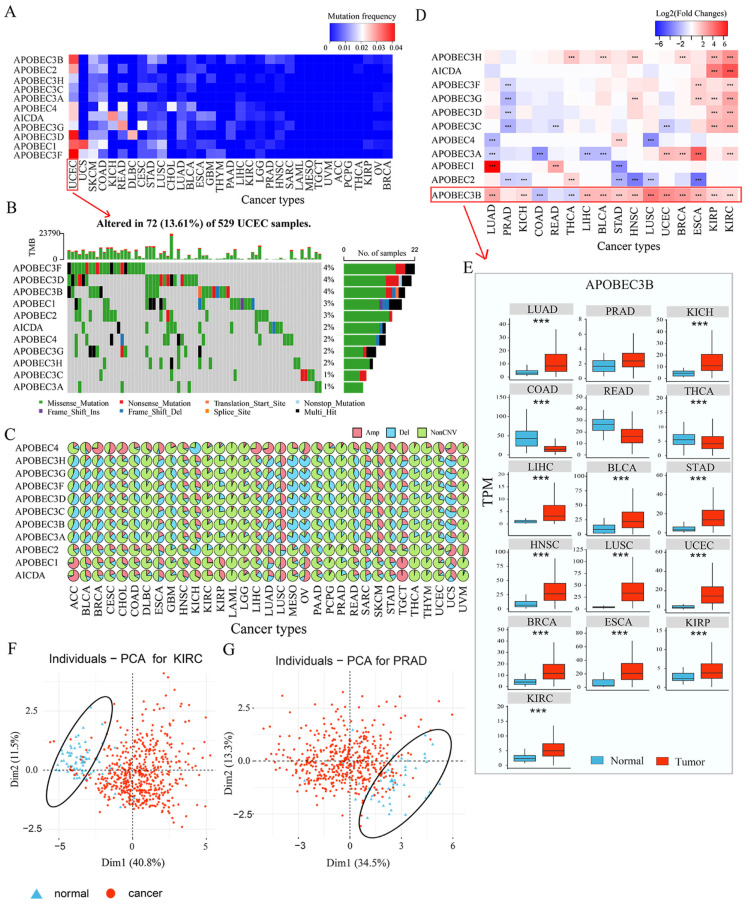
Pan-cancer genetic and expression alterations of APOBEC family. (**A**) The mutation frequency of 11 APOBEC genes across 33 cancer types; (**B**) The mutation frequency of 11 APOBEC genes in 529 UCEC samples. Each column represents individual samples. The upper bar graph shows TMB; the number on the right indicates the mutation frequency in each APOBEC gene. The right bar graph shows the proportion of each variant type; (**C**) The CNV alteration frequency of 11 APOBEC genes across cancer types, Amp: amplification; Del: deletion; (**D**) The gene expression alterations of 11 APOBEC genes in 16 cancer types. The heat map shows the fold changes, with red representing upregulated genes, and blue representing downregulated genes, *** |log2FC| > 1, *p* < 0.005. (**E**) Box plots showing the expression distribution of *APOBEC3B* across tumor and normal samples in 16 cancer types. (**F**,**G**) Principal component analysis for the global expression profiles of 11 APOBEC genes to distinguish tumors from normal samples in KIRC and PRAD, respectively. Two subgroups without intersection were identified, indicating that the tumors and normal samples were well distinguished, based on the expression profiles of APOBEC genes. Tumors were marked with red and normal samples were marked with blue.

To determine the genetic alterations in the APOBEC family in cancer, we first summarized the incidence of somatic mutations across 33 cancer types (Table S1, Supplementary Materials). The overall average mutation frequency of the APOBEC family was low across cancer types, the highest mutation rate was only 4.16%, which was the frequency of *APOBEC3F* in UCEC (Figure 1A). Cancer types with a higher global mutation burden among the 33 cancer types, such as UCEC, also showed a relatively higher mutation rate in the APOBEC family (Figure 1A,B). No mutation of APOBEC genes was observed in several cancer types, such as ACC, PCPG, UVM, TGCT, MESO, and LAML (Figure 1A). We then examined the copy number variations (CNVs) of the APOBEC family across cancer types and found that the incidence of CNV alteration of the APOBEC family in cancer was relatively low (Figure 1C and Table S1, Supplementary Materials). In most tumor types, the CNV alteration frequency of the APOBEC family was less than 50%, while MESO and OV showed a higher CNV variation frequency of deletion, which was more than 70%. The seven members of the APOBEC3 gene cluster shared similar amplifications and deletions, which may be attributed to their location in the genome (Figure 1C). The results suggested that the APOBEC family is relatively stable in the genome of cancer.

To further explore the expression perturbations of the APOBEC family across cancer types, we performed differential expression analysis between normal and cancer samples across the 16 cancer types with at least 10 normal controls. Interestingly, there are extensively significant differences in the expression of the APOBEC family between normal and cancer samples across cancer types (|log2FC| > 1, *p* < 0.05, Figure 1D and Table S1, Figure S1, Supplementary Materials). In KIRP, KIRC, ESCA, HNSC, and BRCA, the expression of most of the APOBEC genes showed significant upregulation in cancer tissues when compared to normal samples, while in PRAD, the expression of the APOBEC genes was significantly downregulated in cancer tissues (|log2FC| > 1, *p* < 0.05, Figure 1D). As consistent with previous studies, we also demonstrated that the APOBEC genes are often deregulated in cancers [3], especially *APOBEC3B* gene. As shown in Figure 1E, the expression of *APOBEC3B* was generally significantly upregulation in cancer tissues compared to normal samples across cancer types, such as BRCA, BLCA, ESCA, LUAD, and STAD. In addition, the deletion frequency of *APOBEC3B* in the CNV alteration was relatively higher in these cancer types (Figure 1C–E). Previous studies have confirmed that the CNV is a partial but not unique factor to regulate mRNA expression [39]. Other features, including RNA methylation and transcription factors, can regulate gene expression [40,41]. Our results also indicate that the genomic alteration (including mutation and copy number variation) may not be the only regulatory mechanism leading to the perturbation of APOBEC expression in cancers. Notably, we found most tumor samples can be distinguished from normal samples based on the global expression of the APOBEC family across cancer types, especially in KIRC, PRAD, LUSC, READ, KICH, KIRP, and BRCA (Figure 1F,G and Figure S2, Supplementary Materials). These results indicated that the occurrence and progression of tumors are closely associated with the expression profiles of the APOBEC family.

**Figure 2:**
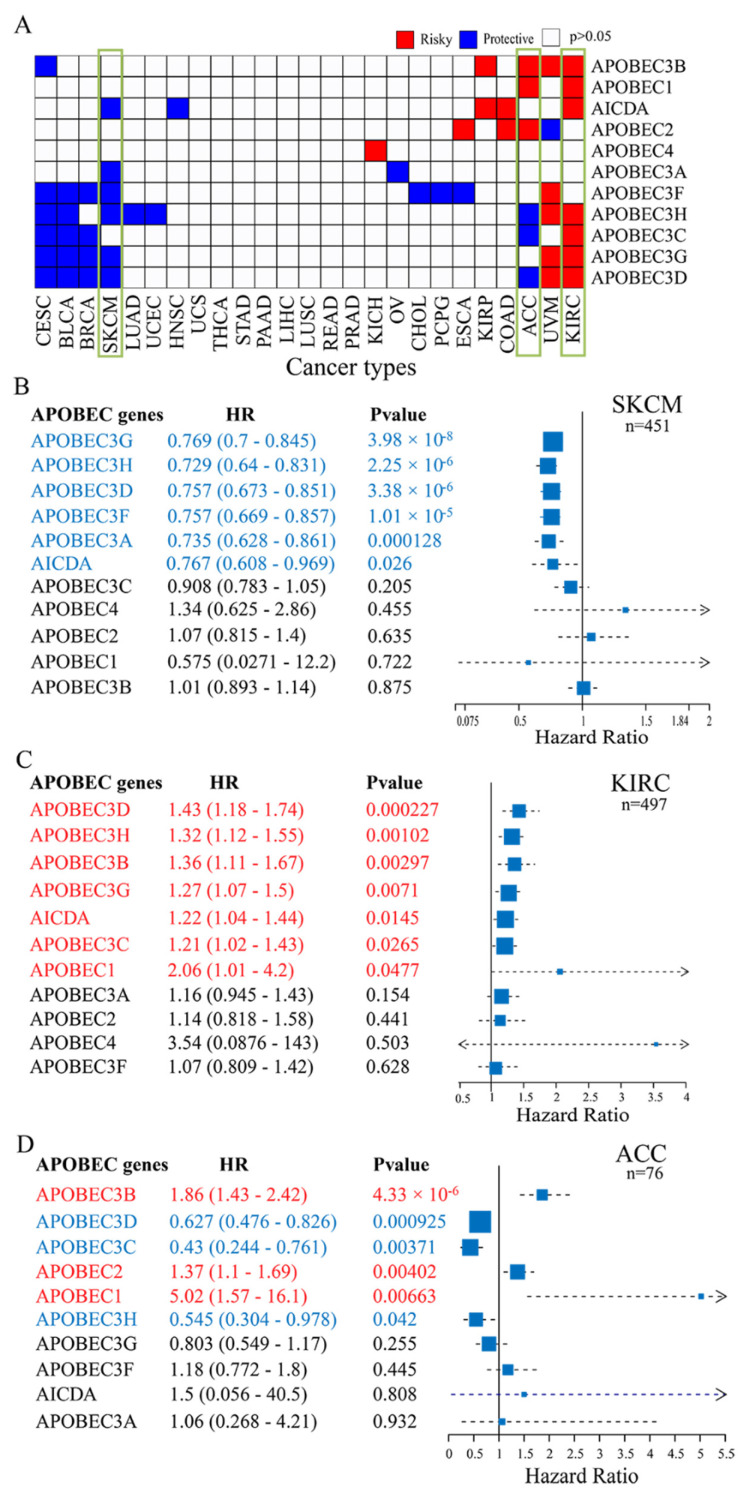
Prognostic value of APOBEC genes across cancer types. (**A**) Summary of the correlation between the expression of APOBEC genes and patient survival across cancer types. Red represents a higher expression of APOBEC gene associated with worse survival, and blue represents an association with better survival. Only *p* < 0.05 are shown; (**B**–**D**) Forest plots show the prognostic value (overall survival) for the 11 APOBEC genes in SKCM, KIRC, and ACC using a univariate Cox regression model, respectively. Hazard ratio > 1 represented risk factors for survival and hazard ratio < 1 represented protective factors for survival.

Using the univariate Cox regression model, we further investigated the association between the expression of the APOBEC family and the survival of tumor patients in 25 cancer types for which overall survival information (including survival status and survival time) was available. As shown in Figure 2A and Figure S1 (Supplementary Materials), the expression levels of the APOBEC genes were significantly associated with prognostic value in 17 cancer types (*p* < 0.05). In 10 of these 17 cancer types, APOBEC genes expression were significantly different between cancer and normal samples (including KIRC, COAD, KIRP, ESCA, KICH, BLCA, BRCA, LUAD, UCEC, and HNSC, |log2FC| > 1, *p* < 0.05, Figure 1D), and in the other 7 cancer types (including UVM, ACC, PCPG, CHOL, OV, CESC, and SKCM), there were lack of sufficient normal samples (*n* < 10) for differential expression analysis. On the other hand, APOBEC expression in six cancer types (including LUSC, STAD, LIHC, THCA, READ, and PRAD) did not showed a significant prognostic association (*p* > 0.05), although its expression was significantly different from that of normal samples (Figure 1D and Figure 2A). Overall, of the 16 cancer types in which APOBEC showed differential expression between cancer and paraneoplasia, only 10 showed a significant prognostic association. Still, of the 17 cancer types with survival differences, 10 had significant transcriptional differences, and it is unknown whether transcriptional differences occurred in the other 7 cancer types due to the differential expression analysis of these 7 cancer types was not performed. These results suggest that when APOBEC is associated with prognosis in a specific cancer type, its expression level tends to differ significantly between cancer and paracancer. In general, differences in protein levels due to differential expression or translation errors caused by DNA or other modifications may trigger survival differences. For the other 7 cancer types, we found that APOBEC showed relatively high mutation frequencies in SKCM and CESC, and extensive CNVs variants in ACC, OV, CHOL, PCPG and UVM (Figure 1A,C). And as shown in Figure S3 (Supplementary Materials), SKCM patients with *APOBEC3B* mutations presented a particularly prominent survival advantage compared to SKCM patients with *APOBEC3B* non-mutations (log-rank *p* < 0.05). Whereas CNV variants in *APOBEC3H* were significantly associated with survival in ACC patients, CNV variants in *APOBEC2* were significantly associated with survival in UVM patients (*p* < 0.05). Therefore, we speculated that the survival differences in these 7 cancer types lacking paraneoplastic samples may also be caused by mutations or gene copy number variants in addition to transcriptional differences. In addition, we observed that in SKCM, many of the APOBEC genes showed beneficial effects, which higher expression of genes was significantly associated with better survival (*p* < 0.05, Figure 2A,B). In contrast, in KIRC, most of the APOBEC genes showed a deleterious effect, which higher expression of genes was associated with worse survival (*p* < 0.05, Figure 2A,C). However, in ACC, the APOBEC genes that were significantly associated with the survival of patients were half-deleterious and half-beneficial (*p* < 0.05, Figure 2A,D). These results show that whether the expression of the APOBEC family has a significant impact on the survival of patients mainly depends on the type of cancer. In addition, we also noted that *APOBEC3F* and *APOBEC3H* were significantly associated with the survival of patients with more cancer types, compared to other family genes (Figure 2A). The above results reveal a highly heterogeneous genetic and expression alteration landscape of the APOBEC family across cancer types, indicating that the expression imbalance is of great significance to tumor development and progression in different cancer contexts, and significantly affects the survival prognosis of patients.

### 3.2. Two Patterns Mediated by APOBEC Family Were Significantly Correlated with Survival

**Figure 3:**
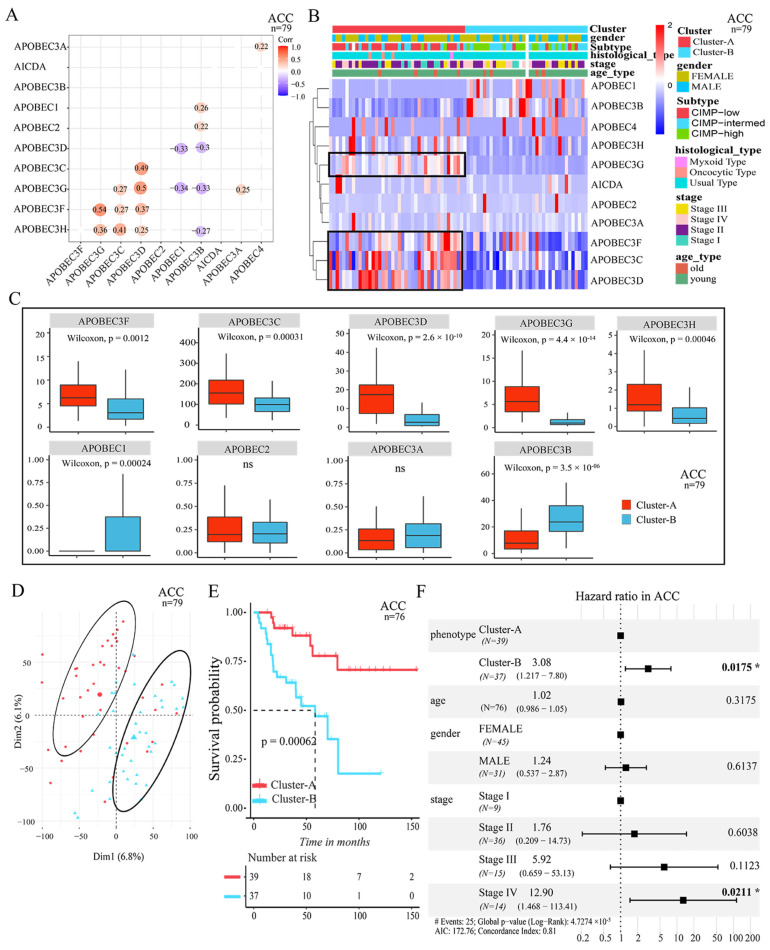
Patterns of APOBEC-mediated stratification and prognosis characteristics of each pattern in ACC. (**A**) Correlations between the expression of 11 APOBEC genes in ACC. A positive correlation is indicated in orange, and a negative correlation is indicated in purple. The color intensity and size of the circle are proportional to the correlation coefficient; (**B**) Unsupervised clustering of 11 APOBEC genes in ACC. The clustering, patient gender, the molecular subtypes, the clinical histological type, tumor stage, and age (old, >65 years old) were used as patient annotations. Each column represented patients and each row represented an APOBEC gene. Red represented high expression of the APOBEC gene and blue represented low expression; (**C**) Box plots showing the expression distribution of APOBEC genes between the two patterns of APOBEC-mediated stratification in ACC; (**D**) Principal component analysis for the transcriptome profiles of the two APOBEC-mediated patterns in ACC, showing a remarkable difference in the transcriptome between different patterns; (**E**) Survival curves of the two patterns of APOBEC-mediated stratification in ACC. Kaplan–Meier plot of overall survival for the two APOBEC-mediated patterns in ACC with prognosis information. The horizontal axis represents the survival time (months), and the vertical axis is the probability of survival. The log-rank test was used to assess the statistical significance of the differences in prognosis between the two pattern tumors; (**F**) Multivariate Cox regression analysis for APOBEC-mediated patterns in ACC is shown by the forest plot. * *p* < 0.05.

To gain a comprehensive understanding of the expression pattern of the APOBEC family at pan-cancer level, a total of >10,000 tumor samples from TCGA 33 cancer types that contained clinical information were selected for further analysis. Each cancer type was analyzed individually. The pairwise correlations between the expression of 11 APOBEC genes showed that the positive correlations were more frequent among *APOBEC3C*, *APOBEC3D*, *APOBEC3F,* and *APOBEC3H*, which means that these genes were co-expressed across cancer types, such as in ACC, SKCM and BRCA cancer samples (*p* < 0.05, Figure 3A and Figure S4A,B, Supplementary Materials). These results suggest that the cross-talk among the APOBEC genes may be important for the generation of different patterns between individual tumors.

Next, using the R package of ConsensusClusterPlus [33], and based on the global expression pattern of the APOBEC family, two patterns of APOBEC-mediated stratification (AMS) in tumor samples were identified across cancer types, respectively. In ACC, for example, the first AMS pattern showed a higher expression of *APOBEC3C*, *APOBEC3D*, *APOBEC3F,* and *APOBEC3G* (namely, Cluster-A), and the remaining tumor samples were characterized by a lower expression of *APOBEC3C*, *APOBEC3D*, *APOBEC3F,* and *APOBEC3G* (namely, Cluster-B) (*p* < 0.05, Figure 3B). The expression level of *APOBEC1*, *AICDA* and *APOBEC4* was generally too low in the tumor samples (e.g., *APOBEC1* in ACC, Figure 3C), so we think that these genes have little contribution to the stratification. Subsequently, principal component analysis was performed to elucidate the difference in transcriptional profiles between the Cluster-A and Cluster-B subgroups (Figure 3D). The results indicated that there was a clear distinction between the two AMS patterns. Notably, we found that the two AMS patterns were significantly correlated with the survival of patients. As shown in Figure 3E, the Cluster-A pattern presented a particularly prominent survival advantage for patients compared to the Cluster-B pattern (log-rank *p* < 0.005). Moreover, by multivariate Cox regression model analysis, which included the factors of patients’ age, gender, and tumor stage status, the results confirmed that the AMS pattern can be a robust and independent prognostic biomarker for evaluating patient outcomes (*p* < 0.05) (Figure 3F).

As consistent with ACC, we also identified the two AMS patterns from other cancer types, respectively, such as in SKCM and BRCA. As shown in Figure S4C,D (Supplementary Materials), the expression regulation of *APOBEC3C*, *APOBEC3D*, *APOBEC3F,* and *APOBEC3G* genes were distinct between the Cluster-A and Cluster-B patterns. Moreover, in these cancer types, the Cluster-A pattern was also proved to have a significant survival advantage for patients compared to the Cluster-B pattern (Figure S5, Supplementary Materials; *p* < 0.05). The results confirmed that, at pan-cancer level, the regulation of APOBEC-mediated stratification can divide tumor samples into two clear distinction patterns, and that the regulation of APOBEC-mediated stratification was significantly correlated with patient survival. In addition, *APOBEC3B* was more widely explored than the other APOBEC genes in the current research. Our results showed that the regulation of *APOBEC3B* in the two AMS patterns for different cancer types was different. In the ACC Cluster-A tumors, the expression of *APOBEC3B* was downregulated, compared to Cluster-B (Figure 3B,C), while in SKCM and BRCA Cluster-A tumors, *APOBEC3B* showed a higher expression (Figure S4C,D, Supplementary Materials). This indicates that, due to the heterogeneity of tumors, the contribution of *APOBEC3B* to the stratification of different cancer types was different.

### 3.3. Distinct TME Infiltration Characteristics in the Two AMS Patterns

**Figure 4:**
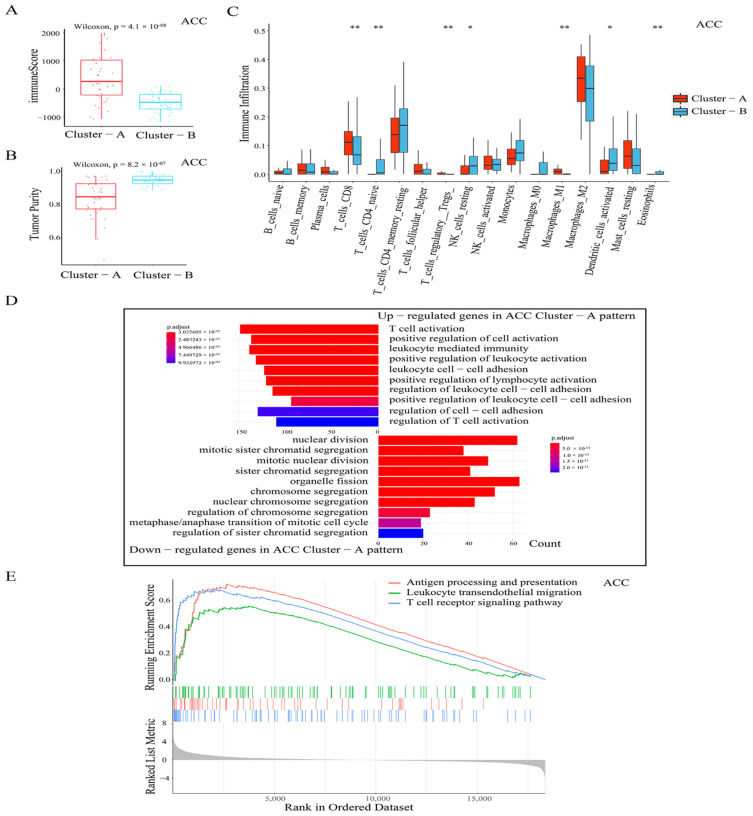
TME cell infiltration characteristics and transcriptome traits in the two patterns of APOBEC-mediated stratification in ACC. Evaluation of immune scores (**A**) and tumor purity (**B**) between the two patterns of APOBEC-mediated stratification in ACC; (**C**) Box plots show the abundance of each TME infiltrating cell in the two APOBEC-mediated patterns in ACC. The asterisks represented the statistical *p* value (* *p* < 0.05; ** *p* < 0.01); (**D**) Functional annotation for DEGs of the two APOBEC-mediated patterns of ACC using GO enrichment analysis. The upper bar plots graph shows the top 10 GO terms results of upregulated genes in Cluster-A pattern tumors in ACC. The lower bar plots graph shows the top 10 GO terms results of downregulated genes in Cluster-A pattern tumors in ACC; (**E**) Gene set enrichment analysis (GSEA) of three immune activation-related pathways enriched in Cluster-A.

A large number of studies have documented the association between TME infiltrating the immune cells and antigens arising from somatic mutations in cancer cells, and the APOBEC family was shown to induce tumor mutations by an aberrant DNA editing mechanism. Therefore, we attempted to comprehensively reveal the integrated role of 11 APOBEC genes in TME infiltration characteristics in pan-cancer. As in ACC, by using the ESTIMATE algorithm [34] to quantify the overall infiltration of immune cells, we found that the Cluster-A pattern exhibited significantly increased immune scores compared with Cluster-B (Figure 4A). By collecting TCGA tumor purity data from previous studies [31], we also observed that the Cluster-A pattern showed remarkably lower tumor purity (cellularity) compared to Cluster-B (Figure 4B). This result indicated that in the Cluster-A pattern tumors, there were more immune cell infiltrations and fewer tumor cells in TME than in the Cluster-B pattern. For a comprehensive assessment of immune cell infiltration, we used CIBERSORT deconvolution [35] to quantify various immune populations using gene expression. As shown in Figure 4C, we found that, compared to the Cluster-A pattern, the infiltration of several immune cell populations was significantly increased in the TME of the Cluster-B pattern, including the T cells CD4 naive, NK cells resting, dendritic cells activated, and eosinophils. There were only three cell populations that showed a significantly higher infiltration in the TME of the Cluster-A pattern compared to the Cluster-B pattern, including T cells CD8, Macrophages M1, and T cells regulatory (Tregs) (*p* < 0.05, Figure 4C). It suggested that the APOBEC-mediated regulation affected only a small part of the TME infiltrating cell types for tumors. Significantly, the higher infiltration of the CD8 T cells in the Cluster-A pattern, which are the cytotoxic T cells with well-recognized significance in anti-tumor immunity, might explain why the Cluster-A patterns have better survival outcomes than Cluster-B, that is, the APOBEC-mediated induction and activation of cytotoxic CD8 T cells in TME, and the consequent intra-tumoral anti-tumor immune response.

For further investigation of the potential biological processes and pathways involved in the molecular heterogeneity between the Cluster-A and Cluster-B patterns, we identified 2574 differential expression genes (DEGs, |log2FC| > 1, FDR < 0.05) between the Cluster-A and Cluster-B subgroups in ACC. Of these, 1714 (log2FC > 1, FDR < 0.05) were upregulated in Cluster-A tumors, and 860 were downregulated in Cluster-A tumors (log2FC < −1 and FDR < 0.05). By GO enrichment analysis for these DEGs, the top 10 GO terms indicated that the upregulated genes were enriched in immune activation-related processes, including T cell activation, positive regulation of cell activation, leukocyte-mediated immunity, and so on; and the downregulated genes were enriched in the cell division-related processes, including nuclear division, chromosome segregation, metaphase/anaphase transition of mitotic cell cycle, and so on (Figure 4D). In addition, the results from the GSEA analysis (Gene set enrichment analysis) also revealed that the Cluster-A pattern was significantly associated with immune activation-related pathways, including antigen processing and presentation, leukocyte trans-endothelial migration, and the T cell receptor signaling pathway (Figure 4E). These results testified that there was a clear distinction in TME infiltration characteristics between the two patterns of APOBEC-mediated stratification in ACC. The Cluster-A pattern was classified as the phenotype of immune activation, characterized by a high immune score, increased infiltration of CD8 T cell, and better survival; the Cluster-B pattern was classified as the low-infiltration immune phenotype, characterized by a low immune score, lack of effective immune infiltration, and poorer survival.

Similar to ACC, the Cluster-A pattern in other cancer types also exhibited significantly increased immune scores and remarkably lower tumor purity compared with the Cluster-B pattern, such as in BRCA, SKCM, PRAD, and KIRC (*p* < 0.05, Figure S6A,B, Supplementary Materials). In particular, the higher infiltration of the CD8 T cells in the Cluster-A pattern was also observed in these cancer types (*p* < 0.05, Figure S6C, Supplementary Materials). Therefore, we speculated that there are two APOBEC-mediated regulation patterns with distinct TME cell infiltration characteristics in pan-cancer. To our surprise, by GO enrichment analysis for the DEGs between the Cluster-A and Cluster-B subgroups in other cancer types, such as in BRCA, we also demonstrated that the upregulated genes, which were higher expressed in the Cluster-A pattern compared to the Cluster-B pattern, were enriched in immunity activation-related processes. The top 10 GO terms included positive regulation of leukocyte activation, leukocyte-mediated immunity, immune response-activating cell surface receptor signaling pathway, and so on (Figure S6D, Supplementary Materials). These results were highly consistent with those in ACC, and confirmed again that the APOBEC family played a non-negligible role in the immune regulation in the tumor microenvironment at pan-cancer level.

### 3.4. Clinical Relevance and Immunotherapy Sensitivity Association of AMS Pattern

**Figure 5:**
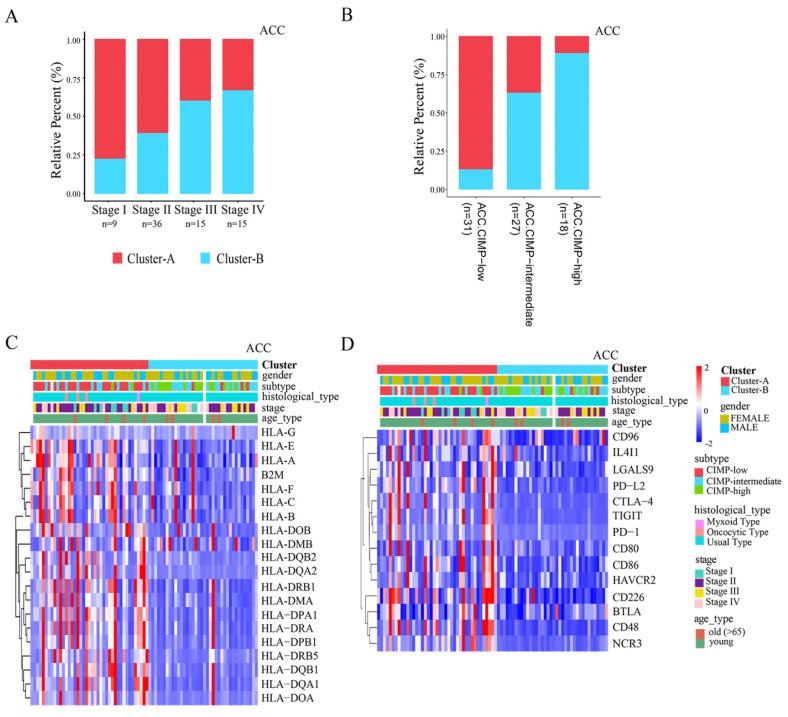
Clinical relevance and immunotherapy sensitivity association of the two APOBEC-mediated patterns in ACC. (**A**,**B**) The proportion of the two APOBEC-mediated patterns in tumor staging and molecular subtypes in ACC. CIMP-high, CpG island hypermethylation; (**C**,**D**) Heatmaps comparing expression profiles of HLA and antigen presenting genes and immune checkpoints molecules between two the APOBEC-mediated patterns.

Observed connections between the AMS pattern and TME features prompted us to explore the clinical significance of these distinct phenotypes. Current staging schemes broadly divide tumors into four stages (or five stages), from stage I to IV (or X). Stage I and II tumors are characterized by being potentially curable by complete resection. Stage III and stage IV (and stage X) tumors are characterized by high invasiveness or metastasis and lower survival [42,43]. Interestingly, when we examined the distribution of APOBEC-mediated stratification in different tumor staging, we found that the Cluster-B tumors were more enriched with the advanced tumors, and the Cluster-A tumors were more enriched in the early tumors. In ACC, for example, the proportion of patients with Cluster-B patterns in stage I—stage IV was 18.2%, 38.9%, 60.0%, and 66.7%, respectively (Figure 5A). In BRCA, the proportion of patients with Cluster-B pattern in stage I to stage X tumors were 59.2%, 57.8%, 61.7%, 75%, and 81.8%, respectively (Figure S7A, Supplementary Materials). It is suggested that the high-stage subtypes were characterized by the APOBEC-mediated pattern with low-infiltration immune. From the above results, we also note that Cluster-B tumors have significantly increased tumor cells in TME compared with Cluster-A tumors (Figure 4B, and Figure S6B, Supplementary Materials). These results confirmed that the APOBEC family plays a crucial role in tumor progression and differentiation.

Recent studies also identified an ACC subtype of CIMP-high (CpG island hypermethylation), which is characterized by rapid recurrent, invasive cancer, and poor survival [44]. Surprisingly, in our study, 88.9% of the CIMP-high tumors were Cluster-B pattern tumors in ACC, while 87.1% of the CIMP-low tumors were Cluster-A pattern tumors (Figure 5B). In addition, previous studies from The Cancer Genome Atlas Network divided SKCM tumors into four genomic subtypes, designated BRAF, RAS (N/H/K), NF1, and Triple-WT [45]. Among these genomic subtypes, SKCM NRAS subtypes were found in the CIMP-high (CpG island hypermethylation) cluster. Similar to ACC, we also found that the RAS subtype contained more SKCM Cluster-B pattern tumors (59.3%), while most BRAF subtype tumors were SKCM Cluster-A pattern tumors (64.2%, Figure S7B, Supplementary Materials). These results indicated that the Cluster-B pattern tumors may have the characteristics of hypermethylation. Moreover, previous studies have revealed that there is a strong correlation between CIMP-high and IDH1 mutation, and the level of genome-wide DNA hypermethylation is significantly increased in IDH1 subtype tumors. In the project of The Cancer Genome Atlas Network, the PRAD tumors were divided into seven molecular subtypes including four subtypes, characterized by cancer-driving gene fusions, or new genes formed by two previously separate genes (ERG, ETV1/4, and FLI1); three of the subtypes are characterized by cancer-driving genetic mutations (SPOP, FOXA1, and IDH1) [46]. Similar to the above results, we also found that the PRAD Cluster-B pattern tumors accounted for 66.7% of IDH1 subtype (Figure S7C, Supplementary Materials). This result confirmed that the APOBEC-mediated pattern with low-infiltration immune was also highly associated with the CIMP-high tumor subtype (CpG island hypermethylation).

The clinical trials and preclinical researches revealed the mechanisms of ICB resistance, including low expression of human leukocyte antigens (HLAs) and immune checkpoints. Considering that the regulation of APOBEC-mediated stratification appears to be associated with the immune microenvironment of the tumor, we explored the sensitivity to immunotherapy between these AMS patterns. As shown in Figure 5C and Figure S7D (Supplementary Materials), the genes encoding HLAs and other antigen-presenting machinery (gene list from George et al. [47]) were expressed at higher levels in the Cluster-A pattern tumors, such as in the ACC and BRCA tumors. Likewise, in these cancer types, we also found that the Cluster-A pattern tumors have a higher expression of many immune checkpoint molecules, including PD-1 (programmed cell death protein-1), TIGIT (T cell immunoreceptor with Ig and ITIM domain), CD80, and CD86, which encode the ligands for cytotoxic T-lymphocyte-associated protein 4 (CTLA-4), and CTLA-4 itself, and so on (Figure 5D and Figure S7E, Supplementary Materials). These results indicated a potential response to treatment with ICB therapy in these patients with the APOBEC-mediated pattern with immune activation.

## 4. Conclusions

In conclusion, we demonstrated the prevalent expression alterations of the APOBEC family across cancer types. Integrated analysis of the APOBEC family revealed an extensive regulatory mechanism by which they affect the tumor microenvironment and the process of tumor oncogenesis and development, and their relationship with patient prognosis in pan-cancer. This systematic analysis of the landscape of molecular alterations, immuno-oncology features, and clinical relevance in the APOBEC family lays a critical foundation for understanding the dysregulation of the APOBEC family. It will also provide insights into the development of related therapeutic targets.

## Data Availability

Not applicable.

## Supplementary Materials

The following supporting information can be downloaded at: https://www.mdpi.com/article/10.3390/cancers14122827/s1, Additional file 1: Supplementary Table S1. APOBEC family genetic and expression alterations across cancer types. Additional file 2: Supplementary Figures S1–S7.

## Abbreviations

CIMP-high	CpG island hypermethylation
ICB	immunological checkpoint blockade
TME	tumor microenvironment
TSG	tumor suppressor gene
FPKM	fragments per kilobase per million
TPM	transcripts per kilobase million
AMS	APOBEC-mediated stratification
DEGs	differentially expressed genes
FDR	false discovery rate
GO	gene ontology
GSEA	gene set enrichment analysis
CNVs	copy number variations
HLAs	human leukocyte antigens
