# Supplementary material for: APOBEC Alteration Contributes to Tumor Growth and Immune Escape in Pan-Cancer
Source: Cancers (Basel). 2022 Jun 8;14(12):2827. doi: 10.3390/cancers14122827 (PMC9221198; doi:10.3390/cancers14122827)
Supplement: Supplementary file 1 [file cancers-14-02827-s001.zip › supplementary Figures S1-S7.pdf]

Supplementary Materials

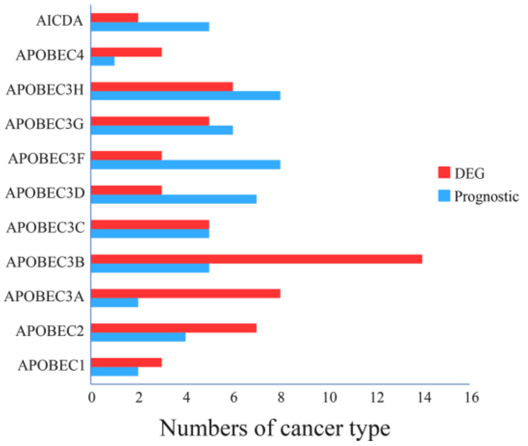

Figure S1 Statistical plot of number of cancer types: Red, number of cancer types with significantly differential in APOBEC gene expression between cancer and normal samples, the significance criteria for determining differential expression in cancer samples was set as |log2FC| > 1 and $p < 0.05$. Blue, the number of cancer types in which expression of APOBEC gene was significantly correlated with cancer prognosis , $p < 0.05$.

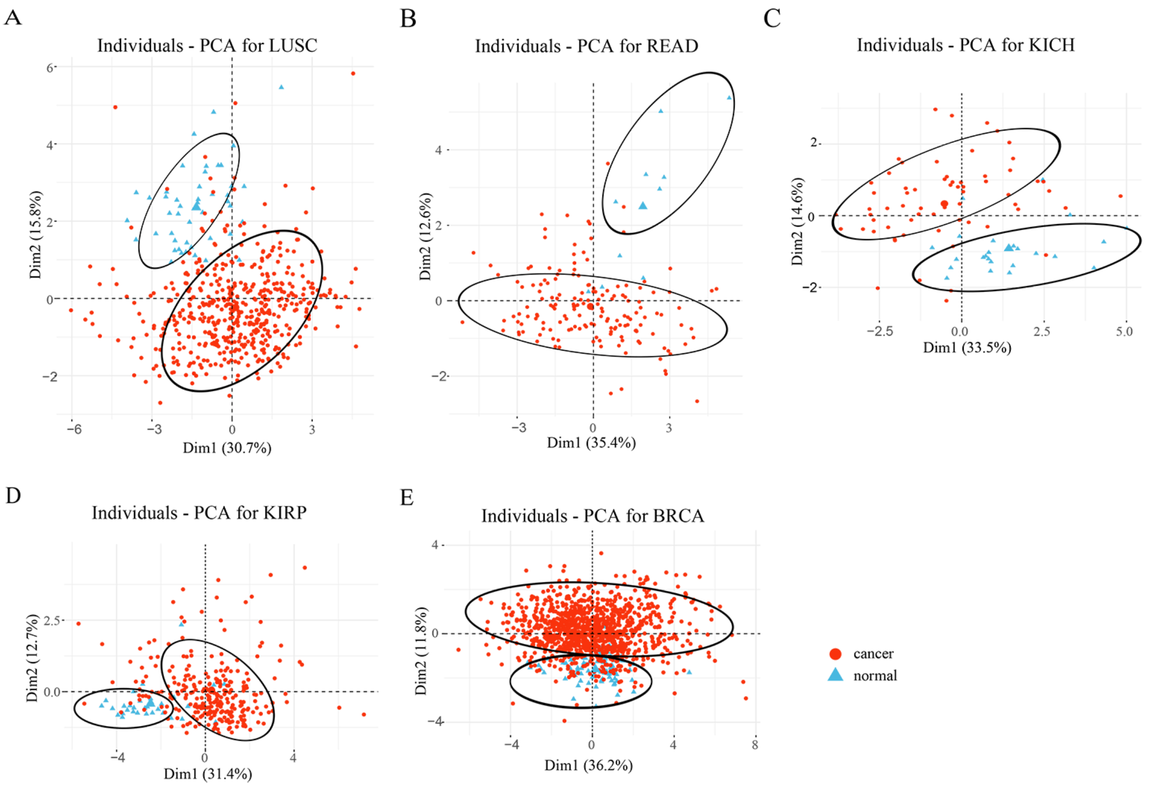

Figure S2 Principal component analysis for the global expression profiles of 11 APOBEC genes to distinguish tumors from normal samples in different cancer types. Such as in LUSC **(A)**, READ **(B)**, KICH **(C)**, KIRP **(D)**, BRCA **(E)**. Tumors were marked with red and normal samples marked with blue.

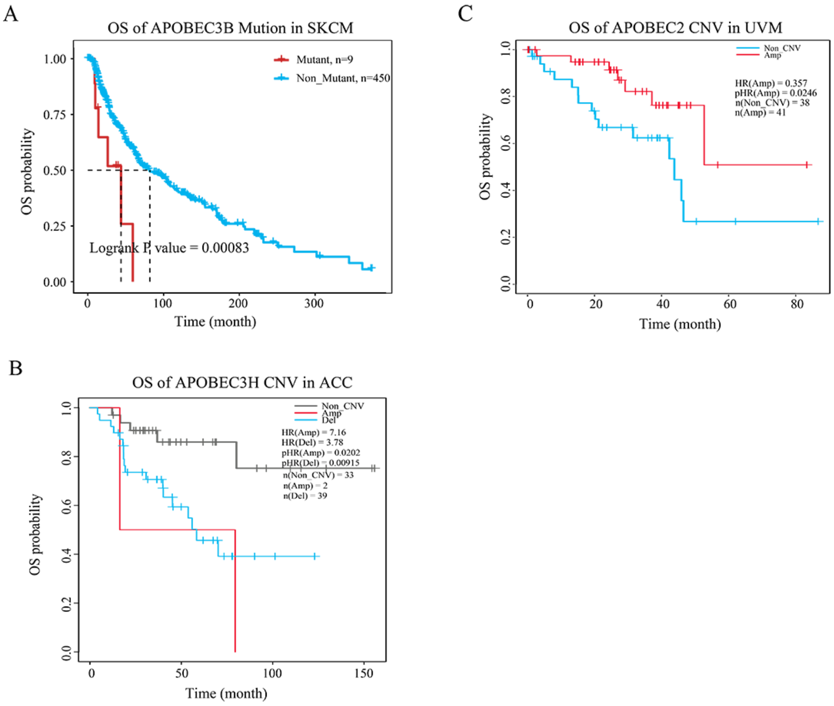

Figure S3 Kaplan-Meier curves of survival for APOBEC mutation or CNVs variation patients. **(A)** Survival curves of *APOBEC3B* mutation in SKCM. **(B)** Survival curves of *APOBEC3H* CNVs variation in ACC. **(C)** Survival curves of *APOBEC2* CNVs variation in UVM.

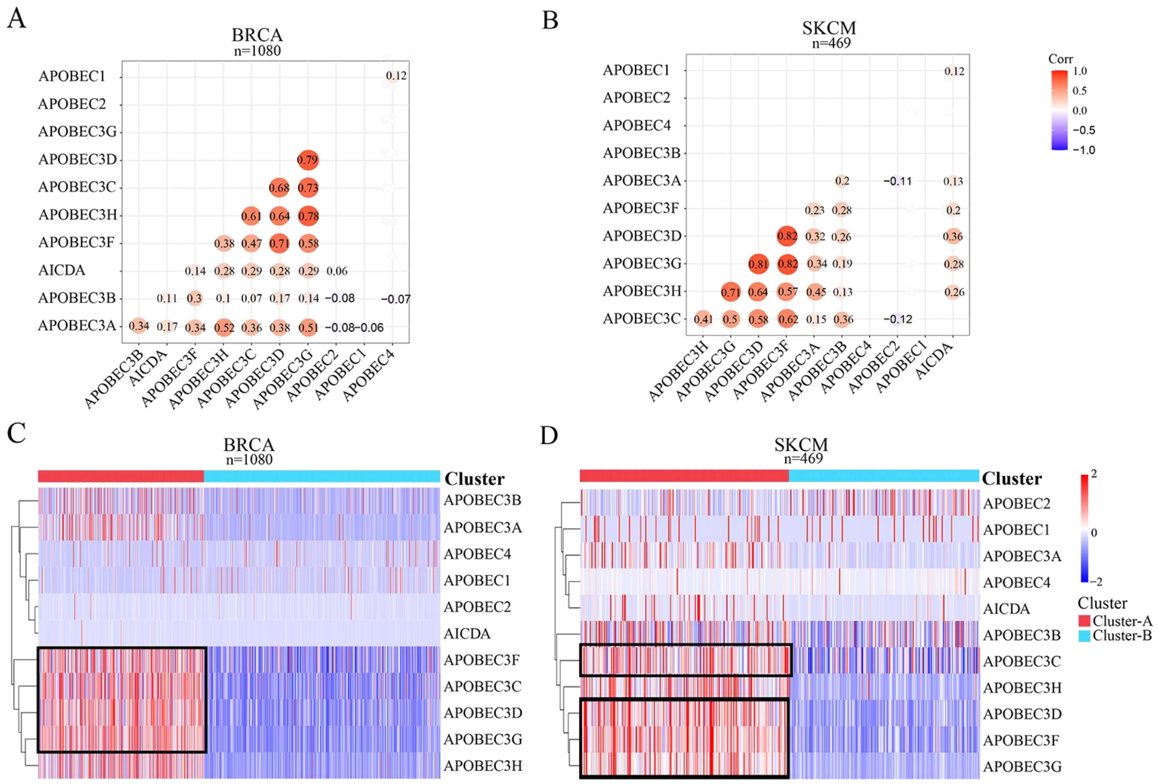

Figure S4 Patterns of APOBEC-mediated stratification in different cancer types. **(A)** and **(B)** Correlations between the expression of 11 APOBEC genes in BRCA and SKCM, respectively. A positive correlation is indicated in orange, and a negative correlation is indicated in purple. The color intensity and size of the circle are proportional to the correlation coefficient. **(C)** and **(D)** Unsupervised clustering of 11 APOBEC genes in BRCA and SKCM, respectively. The clustering was used as patient annotations. Each column represented patients and each row represented APOBEC gene. Red represented high expression of APOBEC gene and blue represented low expression.

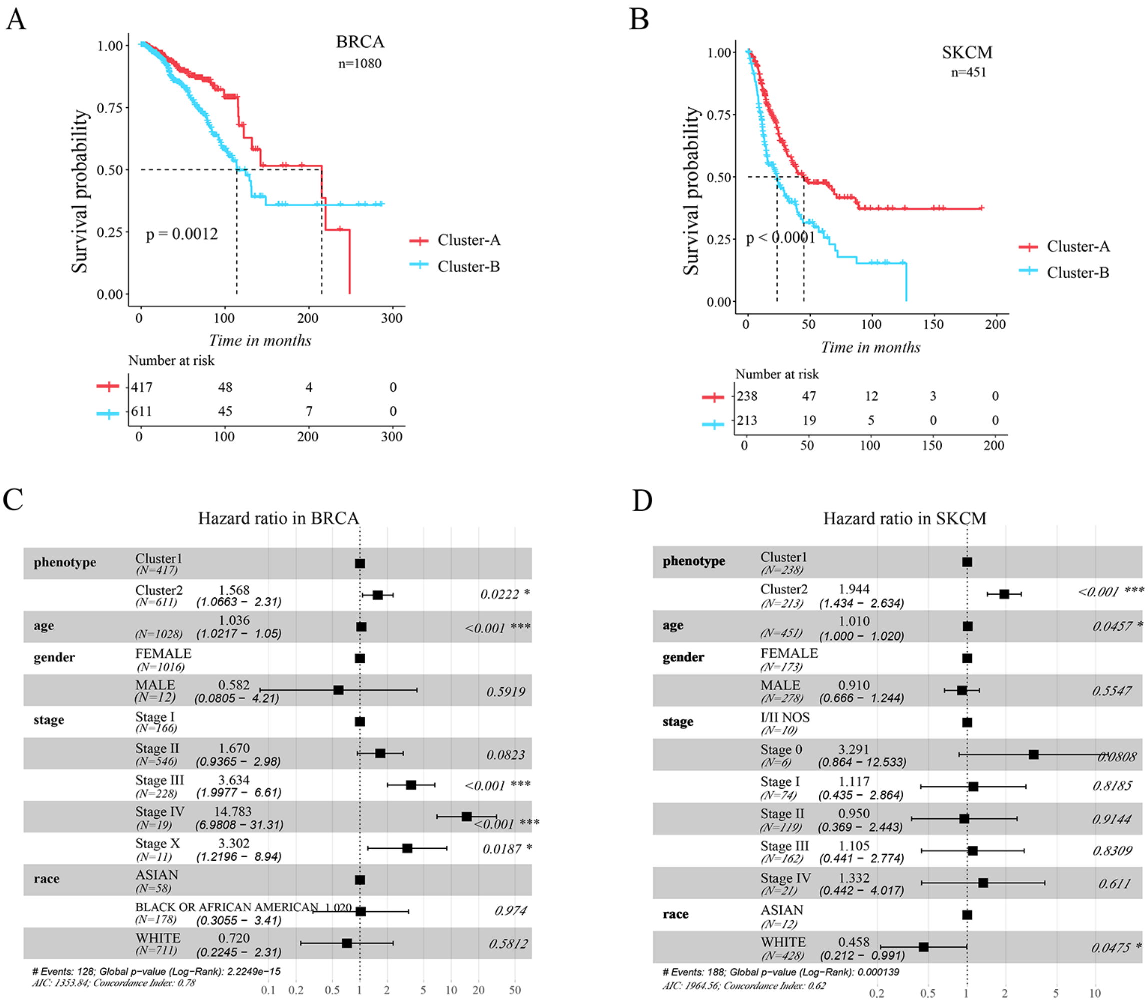

Figure S5 Prognosis characteristics of each APOBEC-mediated pattern in different cancer types. **(A)** and **(B)** Survival curves of the two APOBEC-mediated patterns in BRCA and SKCM, respectively. The horizontal axis represents the survival time (months), and the vertical axis is the probability of survival. The log-rank test was used to assess the statistical significance of the differences in prognosis between the two pattern tumors. **(C)** and **(D)** Multivariate Cox regression analysis for APOBEC-mediated patterns in BRCA and SKCM shown by the forest plot. * $p < 0.05$; *** $p < 0.001$.

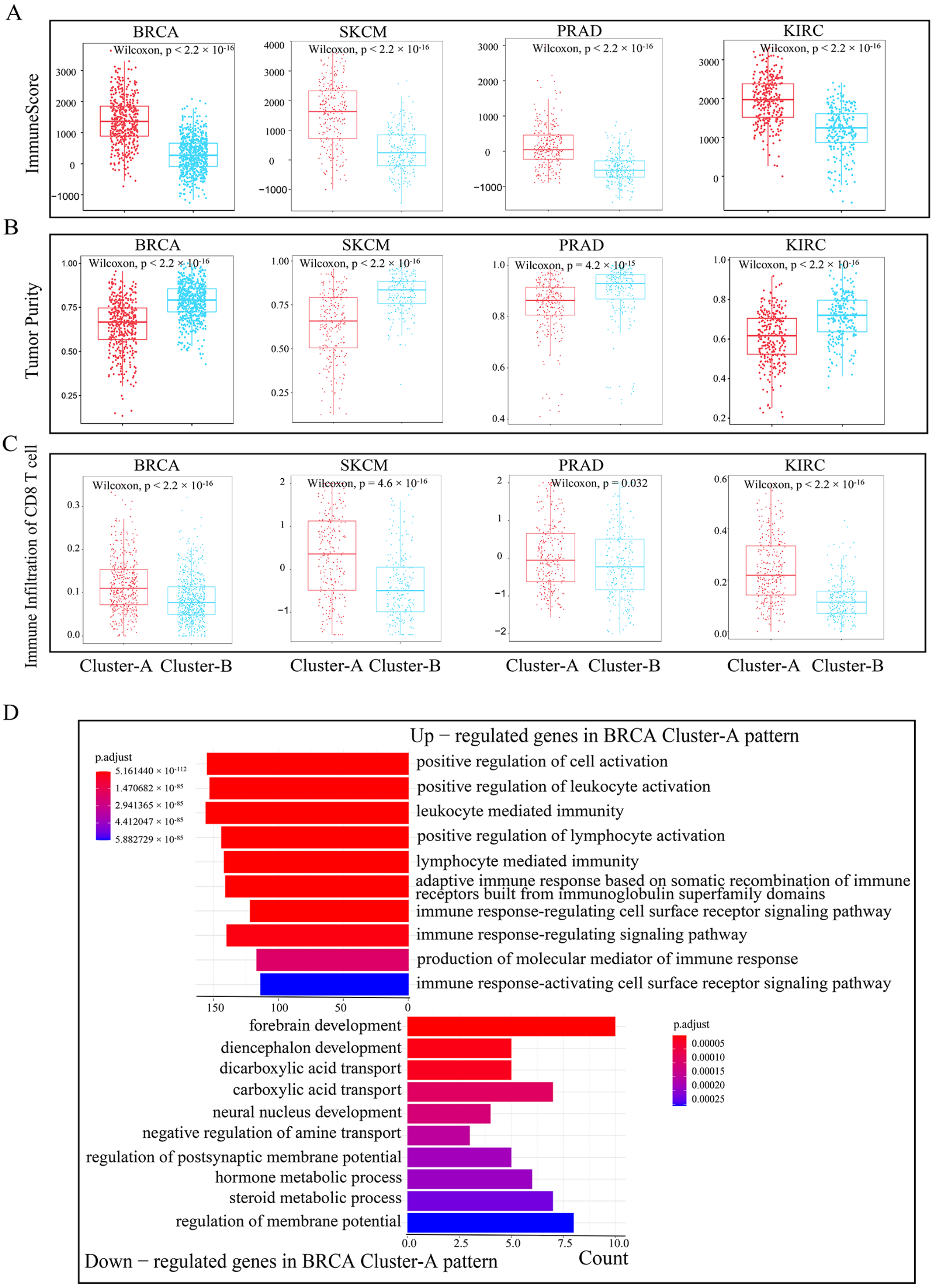

Figure S6 TME cell infiltration characteristics and transcriptome traits in the two APOBEC-mediated patterns in different cancer types. **(A)** Differences in the TME immune scores between two distinct APOBEC-mediated patterns in BRCA, SKCM, PRAD and KIRC. **(B)** Differences in the tumor purity between two distinct APOBEC-mediated patterns in BRCA, SKCM, PRAD and KIRC. **(C)** Differences in the

infiltration of CD8 T cell between two distinct APOBEC-mediated patterns in BRCA, SKCM, PRAD and KIRC. **(D)** Functional annotation for DEGs of the two APOBEC-mediated patterns of BRCA using GO enrichment analysis. The upper barplots graph shows the top 10 GO terms results of up-regulated genes in Cluster-A pattern tumors in BRCA. The lower barplots graph shows the top 10 GO terms results of down-regulated genes in Cluster-A pattern tumors in BRCA.

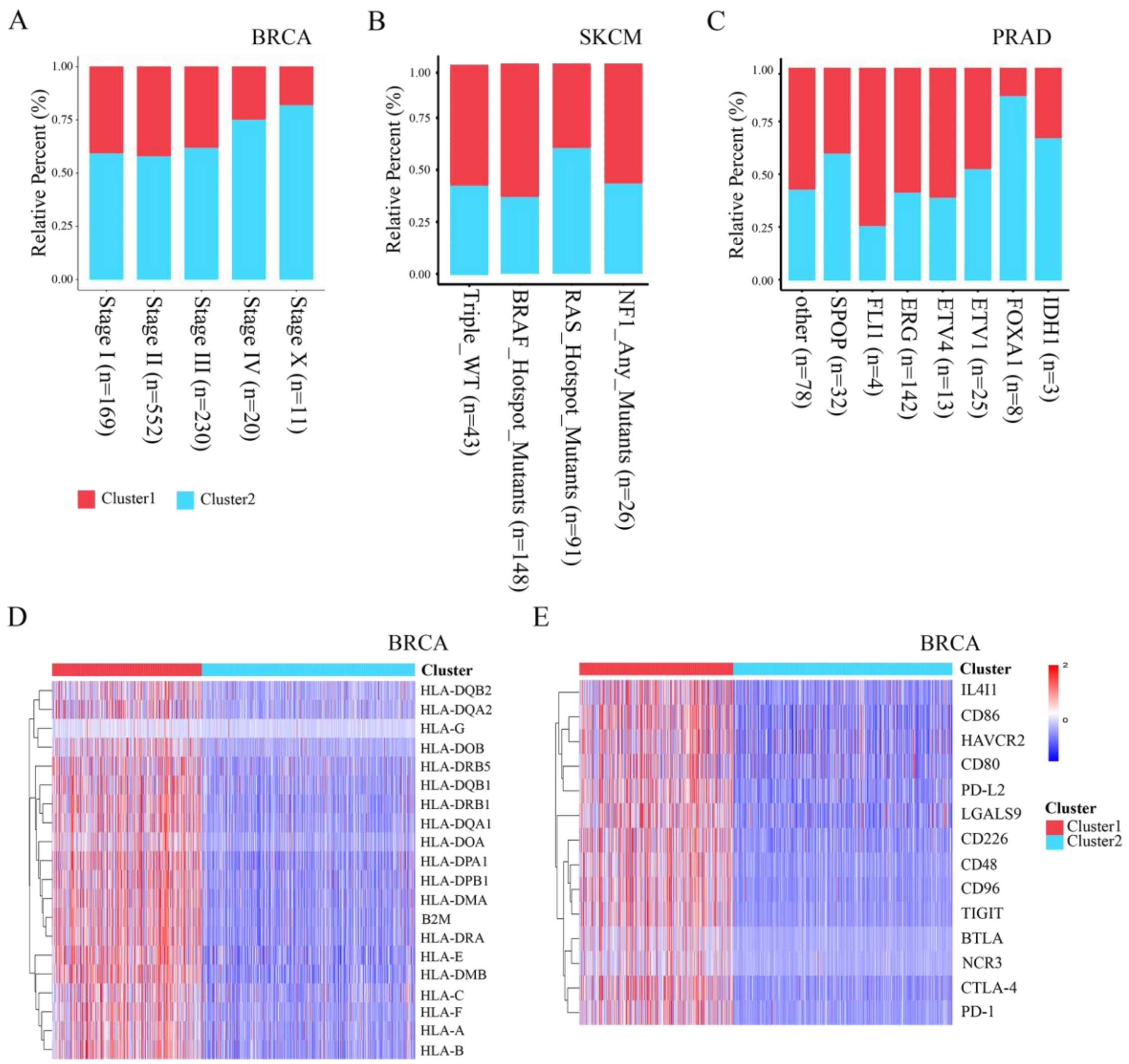

Figure S7 Clinical relevance and immunotherapy sensitivity association of the two APOBEC-mediated patterns in different cancer types. **(A)** The proportion of the two APOBEC-mediated patterns in tumor staging in BRCA. **(B)** and **(C)** The proportion of the two APOBEC-mediated patterns in molecular subtypes in SKCM and PRAD. **(D)** and **(E)** Heatmaps comparing expression profiles of HLA and antigen presenting genes and immune checkpoints molecules between two the APOBEC-mediated patterns in BRCA.
